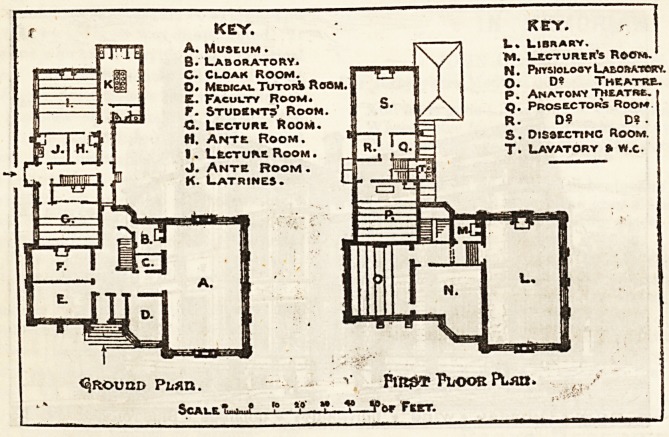# Opening of the New Medical Wing University College, Bristol

**Published:** 1892-11-26

**Authors:** Andrew Clark


### The Institutional Workshop.

### HOSPITAL CONSTRUCTION.

### OPENING OF THE NEW MEDICAL WING, UNIVERSITY COLLEGE, BRISTOL,

By Sir Andrew Clark, Bart., M.D., LL.D., F.R.S.

We regard with special interest the indications of the progressive movement in medical matters at Bristol. This historical old city, which in times past was called, not inappropriately, the city of charities and churches, possesses the oldest provincial hospital but one in the United Kingdom—the Bristol Royal Infirmary, founded in 1735. Its Medical School has existed for several generations, but like most of the provincial schools, it was till recently a comparatively small and unimportant centre of medical education. But a school which has grown to such large proportions, and with which the well-known names of Symonds, Budd, Long Fox, Swayne, and Prichard are so intimately associated, should have been housed in better quarters than the unpretentious buildings that had to do duty heretofore. We congratulate the citizens and medical profession of Bristol on the new buildings which form the highly ornamental medical wing of the University College, which were formally opened on the 16th inst. by the President of the Royal College of Physicians, Sir Andrew Clark, Bart., M.D., F.R.S., in the presence of a large and representative gathering of the medical profession and the citizens of Bristol.

NEW MEDICAL WING UNIVERSITY COLLEGE BRISTOL

In opening the proceedings, the Chairman (the Rev. Dr. Percival) said that that was an important occasion for the city of Bristol. When they saw at that time of the year nearly all the gentlemen who had charge of the health of Bristol gathered together and giving up their time to attend such a gathering, the meeting must be one of unusual importance, and so it was. That day the medical faculty was invited to see their long looked-for, long waited-for, home, and they were there to wish them God speed in the occupation of it.

Mr. Albert Fry, the Chairman of the Council of the University College, explained the present position of the College and Medical School, and why the new building had been brought about. The Medical School had been affiliated to the College, but it had long been felt that its efficiency would be vastly improved if they had a new building. About two years ago they started to carry out the scheme for the erection of a new medical wing; the citizens having come forward and subscribed largely towards the fund. With this help and the aid of some money left to the College, they had been able to put up a building and fit it up, and they stood nearly out of debt. They, however, wanted £200 or £300, more, which the council of the College would be glad to receive to perfect the work. (Hear, hear.)

Sir Andrew Clark, who on rising was loudly cheered, said the occasion which had brought them together was, viewed critically and justly, one of supreme importance to the ancient and illustrious city of Bristol, and they all joined cordially in the expression of the hope that the movement about to be crowned that day might contribute in manifold and in great ways to the life and work, the illumination, and the service of the generation which was to follow the present. To those accustomed to look only upon the surface of things this language would appear exaggerated and unnatural. But to those who penetrated below the surface—who could discern the courses which human thought was pursuing, and the relations which it was establishing between things hitherto far apart and unconnected; who understood that it was only through living ideas that all abiding and fruitful actions were determined, and who measured the value of actions not by immediate material results, but by eventual moral issues—this language would not seem strong enough. They had met to inaugurate the reopening of the Medical School of Bristol. Begun by the doctors, carried on by the people, incorporated by the College, and now crowned, or about to be crowned, as its medical faculty, they celebrated its second birth, and prayed that its second life might be a life of strength, of usefulness, and of honour. The existence of a Medical School in Bristol was of infinitely greater importance to the people than to the medical profession; and the participators in the reorganisation of the Medical School of the people, the College, and the municipality revived that high and true ideal of the civic life which regarded it as a family life. Of the agents actively at work in evolving the just prosperity of a great city such as Bristol, the two chief were the moral agent and the health agent—the moral agent, the true and only source of all abiding good, the neglected remedy of social evils and labour disaffection and unreason; and the health agent, the physical framework which penetrated and influenced all the best that was in man, and all that issued from his thoughts. The health of the individual sensibly influenced, and sometimes even determined and regulated, the amount and quality of his work, and the character and influences of his life. The conditions necessary to the health of the community were not only numerous, but complex, difficult of fulfilment, imperfectly known, and constantly varying. Advancing civilisation brought increasing complexities into the conditions of human life, and if human life was to be continued in all its fulness and fruition they must acquire, not merely a knowledge of those increasingly complex conditions, but also the power of fulfilling, controlling, and re-adjusting them to the varying life and service of mankind. From time to time the problems of existence in fresh conditions must be reconsidered, and renewed endeavours must be made to discover and set forth the most favouring conditions for birth and growth, for education and development, for work and play, for the maintenance of health and resistance to disease, for adjustment to environment and the evolution of fresh capacities and functions, for the prolongation of life and the further physical, mental, and moral development of the race. The investigation of all such subjects, and the proper methods of dealing with them, came under the domain of medicine. In a Medical School they had a centre of medical and general knowledge, and collected together within it a body of highly-educated and selected men, who were striving to discover, teach, and otherwise communicate all that was truest, best, and most useful in the knowledge of the time, and to find out for it the widest and most fruitful applications to the practical business of life. (Hear, hear.) The city of Bristol could not have a great medical school—

and he presumed it would have none but a great medical school—in the midst of it without deriving many and great advantages from its operations and their collateral influences. They would bring to their city the latest and best knowledge in practical medicine and surgery and in the department of public health. They would command the highest and best assistance for the working of their medical charities, and they would be able to procure for themselves and for their families the most experienced and the most skilful service that the age could produce. (Applause.) That was a commercial city, and the continuous and advancing prosperity of such a community depended in a large measure upon the extent to which scientific principles and knowledge could be applied to the manufacturing processes that went on therein. Attached to the Medical School were teachers of chemistry and physics, and it was to the observations and discoveries of such teachers that they owed many of their recent improvements of processes in the arts and manufactures, and most of the economies which had reduced so greatly the cost of producing manufactured articles. It was mainly to the teachers of general science in medical schools that they must look for further progress in that direction, and for relief from some of the increasing difficulties of the labour world, disaffection and unreason. (Hear, hear.) Bristol was happy in having a Medical School which had a justly high reputation—which had produced many distinguished men; which had at various times made substantial additions to their knowledge; which had done admirable work in the relief of suffering, in the cure of disease, and in the improvement of surgical processes; which had educated many successful practitioners in medicine, and which now possessed a teaching staff second to none in the kingdom—a staff which was not only sustaining, but extended the name and fame of the Bristol Medical School. (Applause.) And now the old school, being no longer equal to the educational requirements of the time, and the new school having been built—fitted with all necessary appliances and furniture, with educational instruments, and to a certain extent complete—they were about to enter into the possession. In design, in construction, arrangement, and fittings, the new school was worthy of the highest praise, and might be said to be second to no other provincial school. But it had one defect, and it was so serious a one that unless it were repaired the school could neither adequately discharge its functions nor take rank with any leading medical college. The school had no State licence for experimental research—(cheers)—and, as far as he could learn, it had no intention to apply for one. (Hear, hear, and "No.") He knew—no one knew better—that many estimable and exemplary people, moved by the tenderest feelings and actuated by the highest motives, refused, on the ground that experimental research was both unnecessary and cruel, not only to sanction such a method of inquiry, but to hold any relations with institutions in which it was sanctioned. He had the profoundest sympathy with such persons. He honoured their feelings and respected their motives; but, firmly convinced that they had been prejudiced and misinformed, he strongly dissented from a judgment founded on inaccurate statements and erroneous considerations. (Applause.) In support of his views, Sir Andrew Clark then read his valuable contribution to The Hospital, which appeared in the issue of October 29th. The Medical School having been now built, fitted, and finished, the question naturally and properly arose—how was that admirable but costly building to be maintained in efficient working order? Some people would reply, the building is a doctors' business, why don't the doctors maintain it? But in answer to this he would say that the school was more for the advantage of the public than of the profession, and he held that the public was bound in honour to pay for what was consecrated to its service—(hear, hear)—and in the second place the doctors, speaking quite frankly, were much too poor to be called upon, in a business of that kind, to add to the list of their gratuitous services, and even to pay for services which they rendered to others. Doctors gave more to the public and received less in return than the members of any other profession, and furthermore the work which they did was done at a cost of self-sacrifice, which rendered their lives the shortest of members of any other profession. Strictly speaking, men would ask why the Medical School should not be self-supporting? Well, he did not doubt—indeed, he knew—that some great metropolitan schools had proved not only self-supporting but even moderately remunerative, but he had the gravest doubts if any provincial medical school was ever, from a strictly business point of view, self-supporting. He even doubted whether it was desirable that it should be so, having due regard to its efficiency, place, and influence, and it seemed to him as it had seemed to others ages ago, that the character and importance of its functions made it a just exception to ordinary business rules. The Bristol Medical School could not be made self-supporting, and he should suspect its efficiency and dignity if it were so. The medical faculty could not and ought not to support it. The College could not and ought not to support it; and he saw nothing for it but that the people of Bristol should accept the family idea of the civic life, and that they should make the maintenance of that Medical School, with all its fulness and efficiency, their common duty and constant interest. (Hear, hear.) There were moral interests which would not be repressed, and could not be ignored. The maintenance of the school as he viewed it, with all its high traditions, the beneficent work, its roll of distinguished men, and its splendid promise of future usefulness and honour came within the scope of righteousness. If they believed as he did that the moral idea preceded and determined every true and abiding prosperity, they would regard the maintenance of that school as one of the paramount duties of their citizenship. (Applause.)

The building is a free treatment of English XVIth century work, the exterior faces being composed of local red pennant stone, with dressings of Coombe Down freestone, and is highly creditable to the architect, Mr. F. Bligh Bond, of Bristol.

Over the main entrance is inscribed this very appropriate motto: "Ὦ βίος βραχὺς ἡ δὲ τέχνη μακρή." Passing into the interior we enter a spacious hall with tiled floor and panelled walls, out of which open the museum, a lofty well-lighted room 50 ft. long by 30 ft. broad, the medical tutors' room, a faculty room, students' waiting room, two lecture rooms, preparation rooms, cloak rooms and lavatories.

By a broad pitch pine staircase we ascend to the first floor, which contains a magnificent chamber that is destined to contain the associated libraries of the Bristol Medical School and the Bristol Medico-Chirurgical Society, and capable of receiving 6,000 volumes. This library, which is being handsomely furnished by Dr. E. Long Fox, who also took a leading part in initiating the movement for the Medical School, and is open to the students, and through the Medico-Chirurgical Society, to all medical men of the neighbourhood, and cannot fail to be of inestimable benefit. It has an enriched open hammer-beam roof, with panelled compartments in pitch pine, it is 50 ft. long by 30 ft. wide and about 25 ft. in height to the collar beams.

We may say that the whole building is dadoed and partly ceiled with pitch pine. It is lighted throughout with handsome wrought-iron gaseliers and brackets. Ventilation is performed partly in conjunction with the hot water radiating coils, which admit fresh air, and the exhaust is in connection with the flue from the heating chamber, which is placed in an iron pipe, so as to cause an up current of warm air in the ventilating chamber around it.

The Annual Dinner of the Bristol Medical School, in the evening, was presided over by Sir Andrew Clark, Bart., and about 200 were present, including the Mayor of Bristol, the Very Rev. the Dean, Rev. Dr. Percival, Sir Joseph Weston, M.P., Mr. Charles Townsend, M.P., &c.

The Chairman, in proposing the toast of "The Queen and the Prince and Princess of Wales," said that since their last dinner her Majesty had suffered bereavements and seen some of her most cherished hopes disappointed, and she had had to encounter questions of State fraught with imminent peril. Yet in all these circumstances she had shown that aptitude for business, that insight into affairs, that patience in consideration and thought, and wisdom in council, that fixity of purpose, that loyalty to the Crown, and that respect for the rights of the people which had built up for her, and for her justly, the reputation of being one of the most constitutional sovereigns that ever sat on the English Throne.

The Chairman next proposed "The Bishop and Clergy of the Diocese," and

The Dean, in responding, remarked that it had been said somewhat cynically of some of their more aged bishops that there was one grace they did not know, and that was the grace of resignation. (Laughter.) But on the other hand, it must be largely due to the loving care that their medical advisers had taken of them. (Applause.) Referring to the Church Congress meeting at Folkestone, he said he was ashamed of his cloth when a speaker defined medical men as "scientific devils." (Applause.) He would rather apply to them the words of Sir Walter Scott—

"When pain and anguish wring the brow,
A ministering angel thou."

(Applause.) He could not forget that the cruelties that Miss Cobbe had collected in her book were drawn from the French and German hospitals—(applause)—and it was also fair to remember that all this suffering was to a large extent minimised and mitigated by the administration of anodynes. (Renewed applause.) They might always rely upon his pleading the cause of their hospitals, and he asked them to remember his name was "Dr." Pigou. (Applause.)

The Chairman said that with reference to the term "scientific devils" which the Dean had mentioned as being uttered at the Church Congress, he would remind the clergyman who made use of it of the blessed saying of St. Paul, "Now abideth faith, hope, and charity, but the greatest of these is charity." (Applause.)

Mr. J. Greig Smith proposed "The Past and Present Students," which was acknowledged by Surgeon-Major Peck and Dr. Mortimer Granville for past students, the latter expressing surprise that the profession, being anxious to do homage to Germany, forgot the services of Dr. Swayne, who was at that table, and who in 1849 had actually discovered the comma bacillus or cholera germ, which had been just now rediscovered by a German savant, and the splendid service of Dr. Wm. Budd in reference to typhoid fever. These were things that Bristol men should never forget. (Applause.)

Mr. Nelson Dobson, Senior Surgeon of the Bristol General Hospital, proposed "The Medical Press," referring to the great power and influence that had been exercised by the *Lancet* and the *British Medical Journal*, and now by The Hospital.

Mr. Ernest Hart, responding for the *British Medical Journal*, referred to the high line the medical press endeavoured to take in declining to lend itself to the disgraceful and lying advertisements of pernicious quack remedies which far too readily found admission to the columns of the lay press.

In response to repeated calls, Mr. Burdett, editor of The Hospital, also replied to this toast. He said it was a matter of great satisfaction to himself to find the work of The Hospital so fully recognised by probably the largest gathering of representative members of the medical profession ever assembled in the metropolis of the west. He confirmed the statements of Mr. Hart, and congratulated the Bristol School on the completion of the new buildings, which were referred to as remotely possible when he delivered an address to them only three years ago.

Dr. E. Long Fox, Senior Consulting Physician to the Bristol Royal Infirmary, proposed "The Health of the Chairman."

Several other toasts which we cannot notice in our limited space were duly proposed and honoured.

On Thursday Sir Andrew Clark visited the Bristol Royal Infirmary and the Bristol General Hospital.

We have previously invited attention to the noble medical charities of Bristol, which together afford clinical material for a large body of students. The Royal Infirmary contains 264 beds, and the General Hospital 200, each containing well-organised special departments in every branch of medicine and surgery, and giving opportunities for the students to acquire a practical knowledge of their profession second to none in the kingdom. Bristol is now able to offer students University courses in every faculty, and we look forward to the time when with Birmingham, and we hope Cardiff, its University College will form a part of a Western University, analogous in every respect to the Victoria University of the Northern counties.

## Figures and Tables

**Figure f1:**
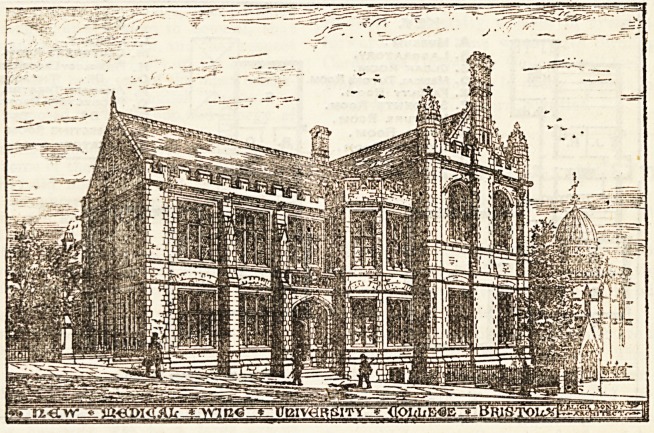


**Figure f2:**